# Pepto-Bismol Tablets Resembling Foreign Bodies on Abdominal Imaging

**DOI:** 10.7759/cureus.2102

**Published:** 2018-01-23

**Authors:** Vahe Shahnazarian, Daryl Ramai, Tagore Sunkara, Vinaya Gaduputi, Andrea Culliford

**Affiliations:** 1 Gastroenterology & Hepatology Fellow, The Brooklyn Hospital Center, Affiliate of the Mount Sinai Hospital. 121 Dekalb Avenue, Brooklyn, Ny 11201; 2 Division of Gastroenterology and Hepatology, Academic Affiliate of the Icahn School of Medicine, Clinical Affiliate of the Mount Sinai Hospital; 3 Internal Medicine/gastroenterology, The Brooklyn Hospital Center, Affiliate of the Mount Sinai Hospital. 121 Dekalb Avenue, Brooklyn, Ny 11201; 4 Department of Gastroenterology and Hepatology, Saint Barnabas Hospital, Bronx, Ny

**Keywords:** imaging, x-ray, ct scan, medication, pepto-bismol, gastroenterology, emergency room

## Abstract

A 28-year-old female presented to the emergency department (ED) with a one-day history of severe, diffuse abdominal pain of sudden onset. In the ED, an abdominal x-ray was done, which showed two hyperdense disc-shaped structures. When her abdominal pain did not subside, the ED performed a computerized tomography (CT) scan of the abdomen and pelvis with contrast, which showed the two previously noted 16 mm disc-shaped objects to actually be located within the cecum. Based on the history and imaging, it was deduced that the two discs were likely to be Pepto-Bismol tablets (Proctor & Gamble Co., Cincinnati, OH). Most medications are radiolucent, but there are some that are radiopaque, including Pepto-Bismol (also known as bismuth subsalicylate). While quite a rare occurrence, it is important to know about since it can lead to unnecessary endoscopy and patient anxiety.

## Introduction

Foreign bodies are not a common finding but are an important and interesting one [1]. In fact, in 2012, a little over half a million emergency department (ED) visits (in all age groups) were attributed to foreign body ingestions [2]. Some require intervention, while others require watchful waiting. In this case, we describe a young female patient who presented to the emergency room with abdominal pain and was found to have foreign bodies on imaging. Her case presents a quandary faced by emergency department physicians and gastroenterologists alike.

## Case presentation

**Figure 1:**
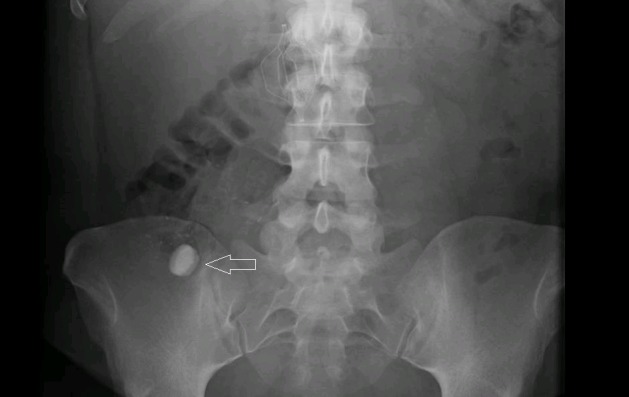
Abdominal x-ray Hyperdense discs at arrow tip.

**Figure 2:**
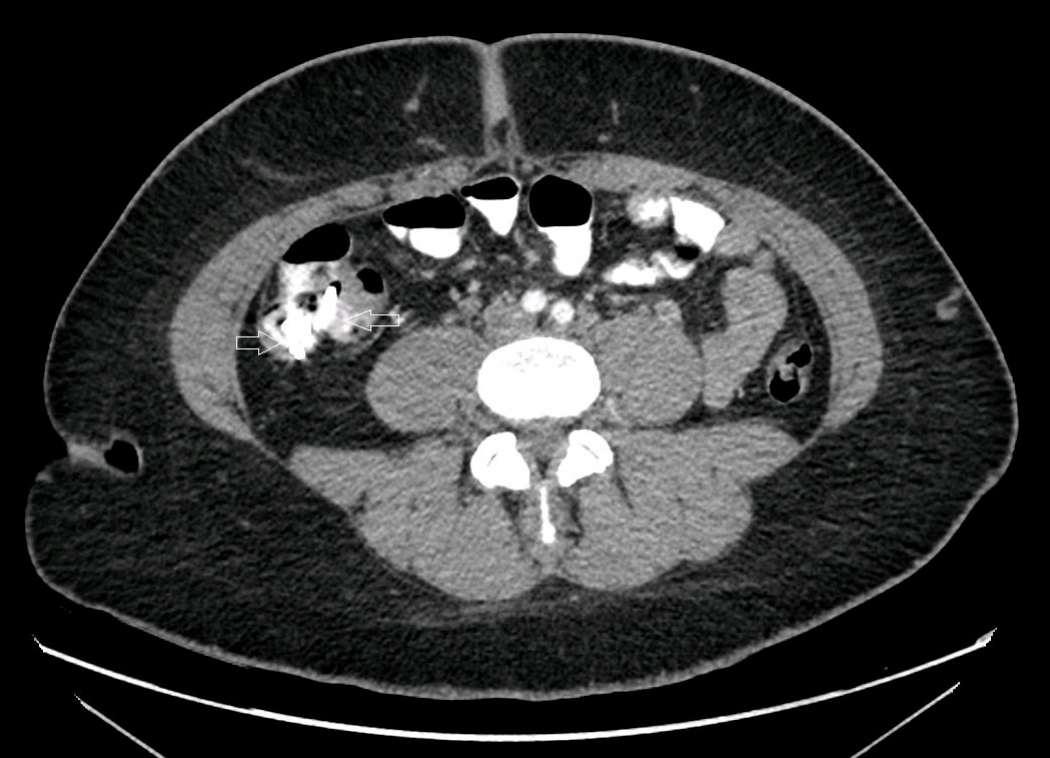
Abdominal computed tomography scan Hyperdense discs at arrow tips, located in the cecum.

A 28-year-old female presented to the emergency department with one day of severe, diffuse abdominal pain of sudden onset. She took two tablets of Pepto-Bismol (Proctor & Gamble Co., Cincinnati, OH) to help with the discomfort, but when that did not work, she went to the ED. In the ED, an abdominal x-ray was done, which showed no abnormality except for two hyperdense disc-shaped structures, probably snaps, projected over the right lower quadrant (Figure 1). When her abdominal pain did not subside, the ED performed a CT scan of the abdomen and pelvis with contrast, which showed the two previously noted 16 mm disc-shaped objects to actually be located within the cecum (Figure 2). The patient adamantly denied swallowing any foreign bodies or placing anything into her rectum. Based on the history and imaging, it was deduced that the two discs were likely to be the Pepto-Bismol tablets. A recommendation for laxatives, serial abdominal exams, and a morning abdominal x-ray was made. The ED was going to work up a gynecological source for the pain, but the patient signed out against medical advice.

## Discussion

While quite a rare occurrence, it is important to know about since it can lead to unnecessary endoscopy and patient anxiety. Most medications are radiolucent, but there are some that are radiopaque, including Pepto-Bismol (also known as bismuth subsalicylate) [1]. When bismuth subsalicylate is ingested in a sufficient amount, it becomes radiopaque enough to be noticeably visible in the gastrointestinal (GI) tract [1]. This typically only rings true for the tablet form, however, as the liquid form is usually too diluted to cause the same effect [3]. While it should not be the only factor in deciding whether or not to perform endoscopy on a patient, knowing about this phenomenon, coupled with a history and physical, will help increase or decrease suspicion and guide management.

## Conclusions

We report the case of a young female with abdominal pain, noted to have foreign bodies on imaging after taking Pepto-Bismol. Although rare, it is quite beneficial to know that certain medications are radiolucent (in their various forms, i.e. liquid, tablet, etc.). This knowledge will assist in guiding therapies and avoiding unnecessary testing and endoscopy.
